# Identification and characterization of *PsDREB2* promoter involved in tissue-specific expression and abiotic stress response from *Paeonia suffruticosa*

**DOI:** 10.7717/peerj.7052

**Published:** 2019-06-12

**Authors:** Huichun Liu, Kaiyuan Zhu, Chen Tan, Jiaqiang Zhang, Jianghua Zhou, Liang Jin, Guangying Ma, Qingcheng Zou

**Affiliations:** Research & Development Center of Flower, Zhejiang Academy of Agricultural Sciences, Hangzhou, China

**Keywords:** PsDREB2, β-glucuronidase, Cis-element, Stress-specific, Histochemical staining, Transgenic engineering

## Abstract

Dehydration-responsive element-binding factor 2 (DREB2) belongs to the C-repeat-binding factor (CBF)/DREB subfamily of proteins. In this study, a 2,245 bp *PsDREB2* promoter fragment was isolated from the genome of *Paeonia suffruticosa*. The fragment was rich in A/T bases and contained TATA box sequences, abscisic acid (ABA)-response elements, and other *cis*-elements, such as MYB and CAAT box. The promoter was fused with the β-glucuronidase (*GUS*) reporter gene to generate an expression vector. *Arabidopsis thaliana* was transformed with a flower dipping method. Gus activity in different tissues and organs of transgenic plants was determined via histochemical staining and quantified via GUS fluorescence. The activity of promoter regulatory elements in transgenic plants under drought, low-temperature, high-salt, and ABA stresses was analyzed. The results showed that the *PsDREB2* gene promoter was expressed in the roots, stems, leaves, flowers, and silique pods but not in the seeds of transgenic *Arabidopsis*. Furthermore, the promoter was induced by drought, low temperature, high salt, and ABA. Hence, the *PsDREB2* promoter is tissue- and stress-specific and can be used in the genetic engineering of novel peony cultivars in the future.

## Introduction

Abiotic stresses, such as drought, cold, salt, and low temperature, were the major limiting factors affecting plant growth and development. During evolution, plants developed a series of adverse response mechanisms. Dehydration-responsive element-binding (DREB) proteins are a class of important signal transduction and transcription factors. A transcription factor specifically binds to the *cis*-acting element of dehydration-responsive element(DRE)/C-repeat (CRT), which is located upstream of the gene promoter, and activates the related stress-induced genes to enhance the resistance of plants (Liu et al., 1998; Yamaguchi-Shinozaki & Shinozaki, 1994). Since the discovery of the *DREB* gene in *Arabidopsis thaliana*, its overexpression in transgenic plants has effectively improved plant tolerance toward multiple stresses (Liu et al., 1998). Thus, the cloning, functional identification, and mechanistic analysis of the *DREB* gene have become hotspots of plant stress resistance genetic engineering (Liu et al., 2000; Ban, Liu & Wang, 2011; Wei et al., 2016; Dong et al., 2017). However, in some cases, ectopic transgene expression occurred and resulted in negative effects such as dwarfism (Kanneganti & Gupta, 2008), delayed growth (Youm et al., 2008), and low yield (Achard et al., 2008). Thus, the extensive study of tissue-specific or stress-inducible promoters and their upstream regulatory factors is essential.

According to their modes and functions, higher-order plant promoters can be divided into three types: constitutive, tissue-specific, and inducible. Constitutive promoters can initiate the expression of target genes in various plant tissues, and gene transcription is persistent without space–time specificity. Currently, in plant genetic engineering, the most commonly used constitutive promoter is *CaMV35S*.This promoter has been widely applied (Okayama et al., 2010; Yang et al., 2015; Zheng et al., 2016; Cai, Song & Zhang, 2016; Azizi et al., 2016; Testroet et al., 2017) but shows some drawbacks. *CaMV35S* induces constant and continuous gene expression during the whole plant growth period in various tissues and organs. This expression feature causes the accumulation of heterologous proteins or metabolites and destroys the original metabolic balance of plants, thereby resulting in serious negative effects on plant growth (Kumpatla et al., 1998; Bhatnagar-Mathur, Vadez & Sharma, 2008). Tissue-specific promoters, also known as organ-specific promoters, are limited to specific organs or tissues and often show the characteristics of developmental stages (Wang et al., 2003). Compared with constitutive promoters, this promoter type has an advantage in plant transgenic application. Exogenous genes can be expressed in specific tissues and at specific developmental stages. However, the exogenous gene expression is not continuous and efficient in all parts and at all stages of the recipient plants. This expression does not affect the normal metabolism of plants, thereby resulting in an unnecessary waste of resources. Recently, tissue-specific promoters have become the focus of plant genetic engineering. Researchers have isolated, cloned, and studied many tissue-specific promoters from different plants (Guo et al., 2010; Koehorst-van Putten et al., 2012; Chen et al., 2013; Zhu et al., 2014; Liu et al., 2015; Xue et al., 2018). The third type of promoter, the inducible promoter, is activated by abiotic or biotic stresses or hormone induction. Currently, *rd29A* is the most widely used inducible promoter in plant genetic engineering (Qiu et al., 2012; Rai et al., 2013; Bihmidine et al., 2013; Lee et al., 2016). This promoter was found in *A. thaliana* and can be induced by abiotic stresses, such as high salinity, low temperature, and drought (Celebi-Toprak et al., 2005; Behnam et al., 2007; Msanne et al., 2011).

The promoter sequence is central to the transcriptional regulation of genes located primarily in the 1,000 bp upstream of the gene transcriptional start site (Kaiser & Batschauer, 1995). Most promoters contain not only a TATA box but also specific *cis*-acting elements related to some functional characteristics. These *cis*-acting regulatory elements consist of approximately 5–25 bp of specific short DNA sequence motifs (Rani, 2007). These sequence elements interact with transcription factors and subsequently activate cascades of genes to improve plant resistance against multiple stresses (Ibraheem, Botha & Bradley, 2010). Hence, an understanding of the *cis*-acting regulatory elements in promoters is essential. Moreover, the mechanism of *DREB* transcription factor promoters can reveal useful information about the genes and signaling networks involved in abiotic stress responses (Sazegari, Niazi & Ahmadi, 2015). Recent studies have focused on the cloning and functional identification of key *cis*-acting elements. However, there are a few reports on the *DREB* promoter. For example, the *GmDREB3* promoter was isolated from the soybean genome; *GmDREB3* gene expression can be maintained at an appropriate level in response to various stresses through the regulation of both positive and negative regulatory motifs (Sun et al., 2008). Moreover, the rice *DREB1B* promoter shows distinct stress-specific responses, and the overrepresented motifs in the promoters of *DREB* genes of rice and sorghum were studied (Gutha & Reddy, 2008; Srivasta et al., 2010). Other *DREB* promoters such as *DREB6* from wheat (Li et al., 2011), *DREB2C* from *Arabidopsis* (Chen et al., 2012), and *DREB1* from buckwheat (Fang et al., 2015) were isolated and studied. The characteristics of partial *DREB* promoters in response to abiotic stresses have been investigated. However, the detailed mechanism of the *DREB* promoter remains unclear. Hence, in-depth research is needed.

Peony (*Paeonia suffruticosa*) is a famous traditional flower in China. With the development of the garden flower industry, increasing attention has been paid to the beautification and medicinal value of peonies. However, environmental factors have limited the planting and application of these flowering plants. To grow beautiful peony flowers south of the Yangtze River in southern China, the directional cultivation of peony varieties with strong resistance has become a very important and urgent need. In our previous study, we found that drought and high salinity could induce the upregulated expression of *PsDREB2*. Overexpression of this gene in *Arabidopsis* remarkably enhanced the resistance of plants against drought and salt (Liu et al., 2016). However, the function of *PsDREB2* in *Arabidopsis* during growth and development and under other signal stimulations is unclear. Hence, the isolation of the *PsDREB2* promoter and the analysis of its functional mechanism are necessary. The upstream promoter sequence of *PsDREB2* from *P. suffruticosa* was cloned to explore the expression patterns of the *PsDREB2* promoter in different tissues and organs of transgenic *Arabidopsis* at different growth stages and under various stresses. Promoter function and activity were visualized through bioinformatic, qualitative, and quantitative analyses.

## Materials and Methods

### Plant materials and growth conditions

*A. thaliana* seeds were surface-sterilized with 1% NaClO for 10 min and washed six times with sterile water. The sterilized seeds were placed on 1/2 Murashige and Skoog (MS) medium with or without 40 mg ⋅ L^−1^ kanamycin for the selection of transgenic and wild-type plants, respectively. The plates were transferred to a plant growth incubator for seed germination under 16 h light (100 mol ⋅ m^−2^⋅ s^−1^)/8 h dark at 22 °C, followed by cultivation under dark conditions for two days.

### Cloning of the *PsDREB2* promoter

Genomic DNA was extracted from the leaf tissues of *P. suffruticosa* using the Total DNA Kit (OMEGA, D3485-01). The 5′-unknown sequence of *PsDREB2* was segregated from the genomic DNA via the Genome Walking Kit (TaKaRa, Code No. 6108) following the manufacturer’s protocol and using four degenerate primers (AP1, AP2, AP3, and AP4) and three specific primers (GSP1, GSP2, and GSP3). The PCR products were purified from 1% agarose gels. The products were then cloned into the pMD-19-T vector (TaKaRa, Dalian, China) and sequenced with the primer pairs F01 and R01. The primer sequences were listed in Table 1.

**Table 1:** Primer sequences used in this study.

Primer names	Primer sequences (5′-3′)
GSP1	CAACAGAAGGGGATCAGCGAAG
GSP2	CTTTGGTTTTACCTCTTGCTCGT
GSP3	CGGATTTCTCATTTTCCCATTTC
F01	TGATTACGCCAAGCTTGCATGCCTGCAGGTCCCCC GCGACGCATGCGCATCCTT
R01	CCGGGGATCCTCTAGAGTCCCCGCTTCCTCGACT AAATATATATATGA
Sequence 1	GCATGCCTGCAGGTCCCC
Sequence 2	GGGGAC
VP 1	CGCAATTAATGTGAGTTAGC
VP 2	CCAACGCTGATCAATTCCAC
DREB1A-F	GTGAGACTCGTCACCCAATATAC
DREB1A-R	GAAATGTTCCGAGCCAAATCC
GUS-PF	GAATACGGCGTGGATACGTTAG
GUS-PR	GATCAAAGACGCGGTGATACA
CBF1-PF	GAGACGATGGTGGAAGCTATTT
CBF1-PR	AGCATGCCTTCAGCCATATTA
RD29A-PF	GTGAGGCATCAGAAGAGGATAAA
RD29A-PR	GATGAGAAAGTTCCGGTGAGAA
RD29B-PF	CCAGAACTATCTCGTCCCAAAG
RD29B-PR	GAAGCTAACTGCTCTGTGTAGG
TUB2-PF	GGCCTTGTACGATATTTGCTTC
TUB2-PR	TCGGAGGTCAGAGTTGAGTTGA

### Bioinformatics analysis of the promoter sequence

The *PsDREB2* 5′-upstream promoter regions were scanned for the presence of *cis*-acting elements using the online program PlantCARE (https://bio.tools/plantcare). The regulatory elements were analyzed by using the PLACE program (http://www.dna.affrc.go.jp/PLACE).

### Construction of the promoter-GUS reporter plasmid

With the genomic DNA of peony as a template, a 2,245 bp sequence upstream of the translational start codon of the *PsDREB2* gene was cloned by PCR with the primers F01 and R01. The cloned *PsDREB2* promoter sequence was digested with *HindIII* and *XbaI* restriction enzymes. Target fragment 1 was recovered by gel extraction and referred to as Insert DNA. The *pBI121* vector with the *GUS* gene was also digested with *HindIII* and *XbaI* restriction enzymes. Target fragment 2 was recovered by gel extraction and referred to as Vector DNA. Insert DNA and Vector DNA were ligated with the In-Fusion HD Cloning Kit (TaKaRa, Code No. 639633) to construct the *pBI-PsDREB2:: GUS* vector.

### *Arabidopsis* transformation

The recombinant plasmid was introduced into *Agrobacterium tumefaciens GV3101* through electrotransformation and was subsequently used to transform *Arabidopsis* plants via a flower dipping method as reported previously (Clough & Bent, 1998). The transformants were selected by planting the seeds on 1/2 MS plates containing 40 mg ⋅ L^−1^ kanamycin. The positive transgenic plants were verified via genomic PCR with *GUS* gene primers and the corresponding promoter-specific primer pairs VP1 and VP2. The plasmid pBI121 with the *GUS* gene was used as a positive control. Transgenic seedlings during different developmental stages and in different tissues, including roots, stems, leaves, flowers, and fruits, were collected for spatiotemporal expression assays via GUS histochemical staining and fluorometric assays.

### Stress treatments

Twenty-five-day-old transgenic *Arabidopsis* plants (T3) were treated with ABA, NaCl, PEG6000, and low temperature to characterize the induced activities of the *PsDREB2* promoter in response to different abiotic stresses. The roots were subjected to 100 µmol ⋅ L^−1^ ABA, 300 mmol ⋅ L^−1^ NaCl, 10% PEG6000, and 4 °C for 2 h. Some of these treated plants were immediately collected for GUS staining assays; the other plants were frozen in liquid nitrogen and stored at −80 °C for GUS quantitative assays and real-time PCR. Water-treated and wild-type *Arabidopsis* plants served as the controls.

### GUS activity assay

Histochemical localization of GUS activity was performed using the GUS Staining Kit (Beijing, RTU4032). Transgenic *Arabidopsis* plants or tissues were vacuum infiltrated in a solution containing 50 × X-gluc (1 volume) and GUS staining buffer (50 volumes) for several minutes and incubated overnight in the dark at 25 °C. The incubated plants or tissues were then rinsed with 25%, 50%, 75%, and 95% ethanol. The plants or tissues were vibrated gently on a shaker incubator for 20 min at 25 °C prior to microscopic examination.

Quantitative measurements of GUS activity were carried out using the Plant GUS ELISA Kit (Cat. No. JL13690; Beijing, China). Analyst 1.5 software (Applied Biosystems) was used for data acquisition and processing. The linearity in ionization efficiencies was verified by analyzing a dilution series of standard mixtures. *GUS* activities were quantified relative to the signal of their corresponding internal standard. For the quantification of GUS activity, an internal standard was used, and an experimentally determined response factor of 1 was applied.

### RNA isolation and real-time PCR

Total RNA was isolated from *Arabidopsis* seedlings using the miRNeasy Mini Kit (QIAGEN, 217004). Reverse transcription was performed by using the PrimeScript RT Reagent Kit with gDNA Eraser (TaKaRa, RR047A) according to the manufacturer’s instructions. Quantitative reverse transcription-PCR (qRT-PCR) was performed on the Mastercycler^®^ ep realplex Real-time PCR System (Eppendorf, Germany) according to the manufacturer’s instructions. The PCR cycle conditions for qRT-PCR were set to default: 50 °C for 2 min and 95.0 °C for 2 min, followed by 40 cycles at 95.0  °C for 15 s, 60.0 °C for 30 s, and 72.0 °C for 30 s. *AtDREB1A* and *GUS* genes were amplified with the specific primers At-PF/PR and GUS-PF/PR, respectively. The mRNA levels for each cDNA probe were normalized with respect to the *Tub2* mRNA level. The relative gene expression was analyzed using the 2^−ΔΔ*Ct*^ method (Livak & Schmittgen, 2001).

### Statistical analysis

Data are shown as the means with standard errors of three independent biological samples. GraphPad Prism 5 software was used for statistical analysis. In all graphs, the error bars indicate the standard deviation.

## Results

### Cloning and bioinformatic analysis of the *PsDREB2* promoter sequence

**Figure 1:**
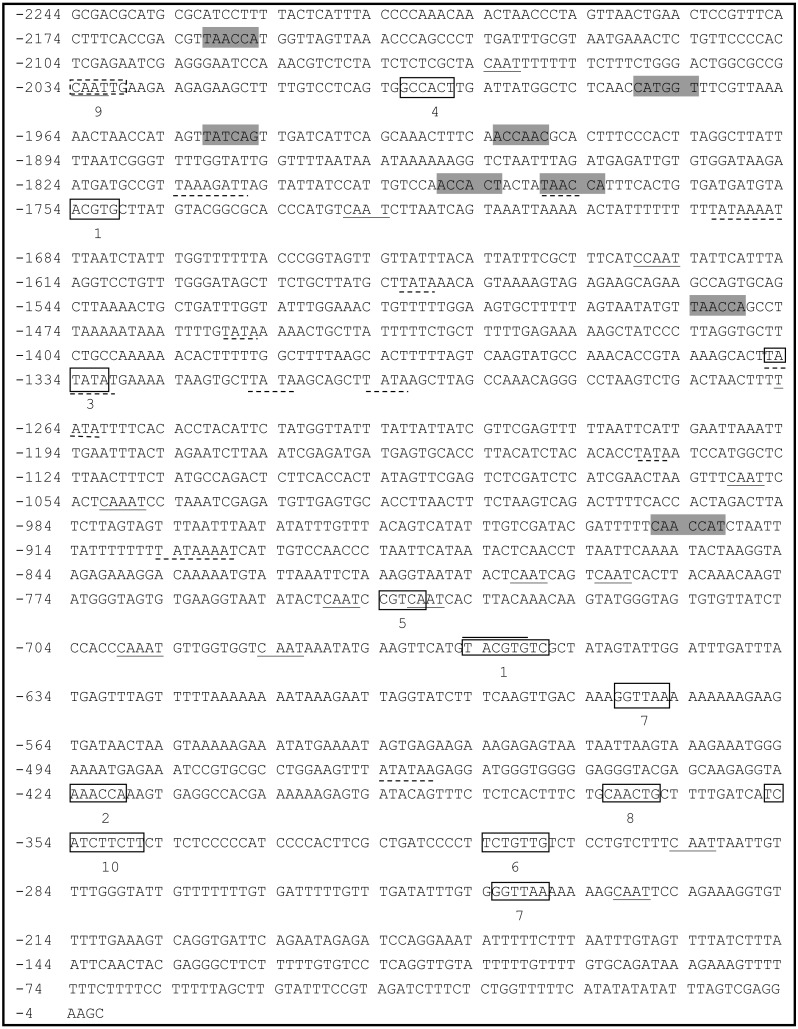
Analysis of the *PsDREB2* promoter sequence using the PlantCARE database. Underlined sequence: CAAT-box; Overlined sequence: G-box: light-responsive element; Gray sequence: MYB recognition site; Underlined with dotted line: TATA-box. 1: ABRE: ABA-responsive element; 2: ARE: anaerobic induction element; 3: AT-TATA; 4: CAT-box: meristem expression element; 5: CGTCA motif: MeJA-responsive element; 6: GARE motif: gibberellin-responsive element; 7: GT1motif: light-responsive element; 8: MBS: drought-inducible element; 9: MYC recognition site; 10: TCA: SA-responsive element.

A 2,244-bp fragment located upstream of the ATG start codon of *PsDREB2* was amplified using the genome walking method (Fig. 1). The promoter sequence was analyzed by using PLACE and PlantCARE web tools. The results of the bioinformatics analysis revealed that the *PsDREB2* promoter contains numerous *cis*-acting elements (Table 2). Fifteen TATA boxes, which are required for critical and precise transcription initiation, were found at various positions covering the whole promoter upstream sequence. Fifteen CAAT boxes, which were responsible for the tissue-specific promoter activity, were found at the position from −2,064 to −274 bp. The CAAT box was responsible for meristem expression and was found at −1,997 bp. Three light-responsive elements, such as one G box and two GT1 motifs, were predicted in the promoter. Many stress-responsive elements were predicted, such as the anaerobic-responsive element (ARE) involved in anaerobic induction, the MYB recognition sites involved in drought and ABA signals, and the MYC recognition site involved in drought, ABA and cold signals. Furthermore, hormone-responsive elements, such as the ABA response element (ABRE) motif in response to ABA, the CGTCA motif in response to methyl jasmonate (MeJA), the GA-responsive element (GARE) motif in response to gibberellin, and the TCA motif in response to salicylic acid (SA), were found in the promoter sequence.

**Table 2:** Prediction *cis*-acting elements of *PsDREB2* promoter using PLANT CARE database analysis.

Site name	Element core sequence	Element number	Function
ABRE	ACGTG	4	cis-acting element involved in the abscisic acid responsiveness
ARE	AAACCA	1	cis-acting regulatory element essential for the anaerobic induction
CAAT-box	CAAT	15	common cis-acting element in promoter and enhancer regions cis-acting regulatory element related to meristem expression
CAT-box	GCCACT	1	
CGTCA-motif	CGTCA	1	cis-acting regulatory element involved in the MeJA-responsiveness
G-Box	TACGTG	1	cis-acting regulatory element involved in light responsiveness
GARE-motif	TCTGTTG	1	gibbreellin-responsive element
GT1-motif	GGTTAA	2	light responsive element
MBS	CAACTG	1	MYB binding site involved in drought-inducibility
MYB	TAACCA	8	response to drought and ABA signals
MYC	CAATTG	1	response to drought, ABA and cold signals
TATA-box	TATAA	15	core promoter element around -30 of transcription start
TCA	TCATCTTCAT	1	cis-acting element involved in salicylic acid responsiveness

### Spatiotemporal expression of the *PsDREB2* promoter in *Arabidopsis*

**Figure 2:**
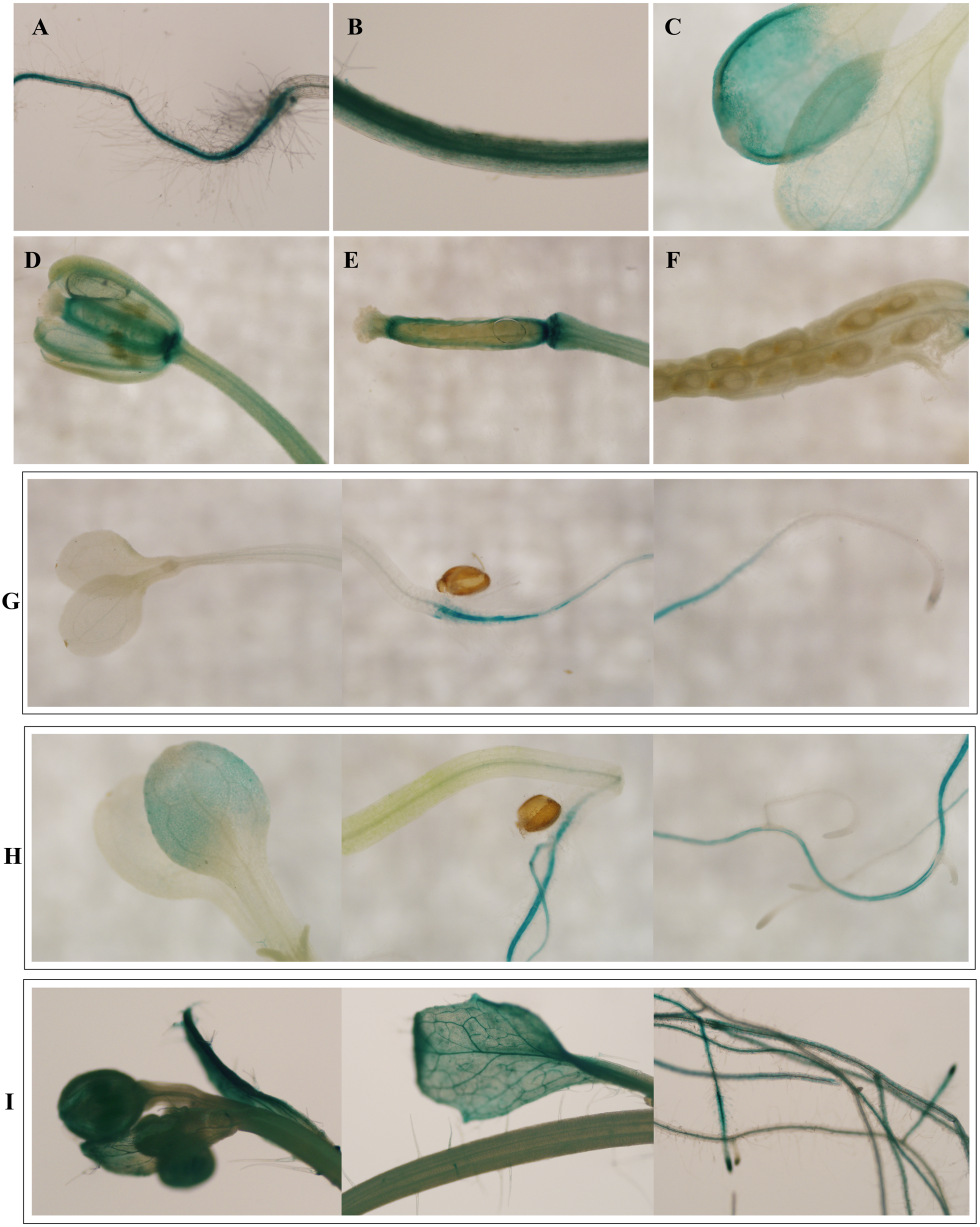
Spatiotemporal expression pattern of the *PsDREB2* promoter in transgenic *Arabidopsis* plants. Transgenic *Arabidopsis* was analyzed by using histochemical GUS staining assays. (A–F): Tissues. (A) Shoot; (B) Stem; (C) Leaf; (D) Flower; (E) Pod; (F) Seed. (G) 5-day-old seedlings. (H) 10-day-old seedlings. (I) 25-day-old seedlings.

**Figure 3:**
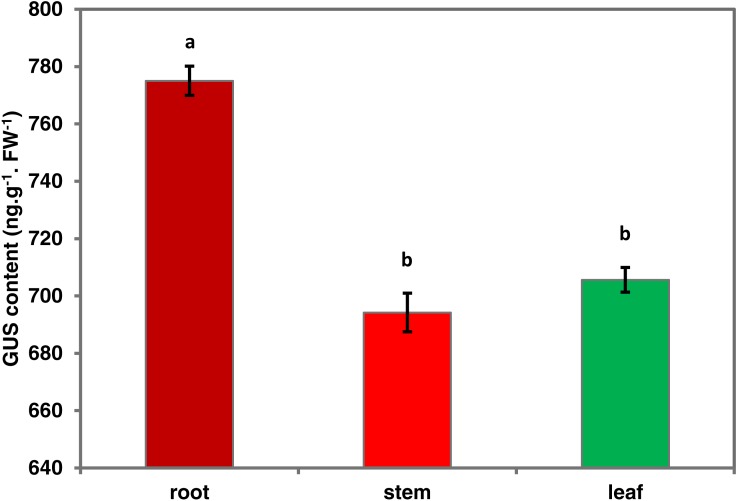
Quantitative GUS activity in the different tissues of transgenic *Arabidopsis* plants. Roots, stems and leaves were isolated from 25-day-oldtransgenic T3 *Arabidopsis* plants with the *PsDREB2* promoter and assayed for GUS quantitative activity. Mean values are expressed in ng protein per g fresh weight. Data are presented as the means of three replicates with ±SDs shown by vertical bars. Different letters of a and b differ significantly by one-sided paired *t* test at *P* < 0.05.

To investigate the spatiotemporal expression patterns of the *PsDREB2* promoter, the *PsDREB2*-2,245 bp-driven *GUS* reporter gene was monitored during plant developmental stages and in different tissues by using histochemical staining in T3 transgenic *Arabidopsis*. *GUS* expression was detected in the roots, leaves, stems, flowers, and silique pods but not in the seeds (Fig. 2A). In 5-day-old *Arabidopsis*, strong *GUS* expression was detected at the maturation and elongation zones of primary roots; however, sporadic staining at only the meristematic zone and no staining in the leaves and stems were observed (Fig. 2B). In 10-day-old plants, *GUS* expression was present in the roots and leaves (Fig. 2C). However, in 25-day-old plants, *GUS* expression with uneven distribution was detected throughout the whole plants (Fig. 2D). GUS activity was higher in the roots and leaves than in other tissues. In the leaves, especially in the young leaves, GUS staining was more evident in veins and stomas than in mesophyll tissues. GUS activity was intense in the elongation zone of primary roots and most of the whole lateral roots. The stem hairs and vascular tissues were intensively stained. The quantitative examination of 25-day-old transgenic T3 *Arabidopsis* plants was conducted to calculate GUS activity in the roots, stems, and leaves. The assay results showed that GUS activity was highest in the roots, followed by the leaves and then the stems (Fig. 3), which corroborated the above results. During all developmental periods, GUS activity was not detected in the corresponding tissues of wild-type (WT) *Arabidopsis* plants.

### Characteristics of the *PsDREB2* promoter in response to various abiotic stresses

**Figure 4:**
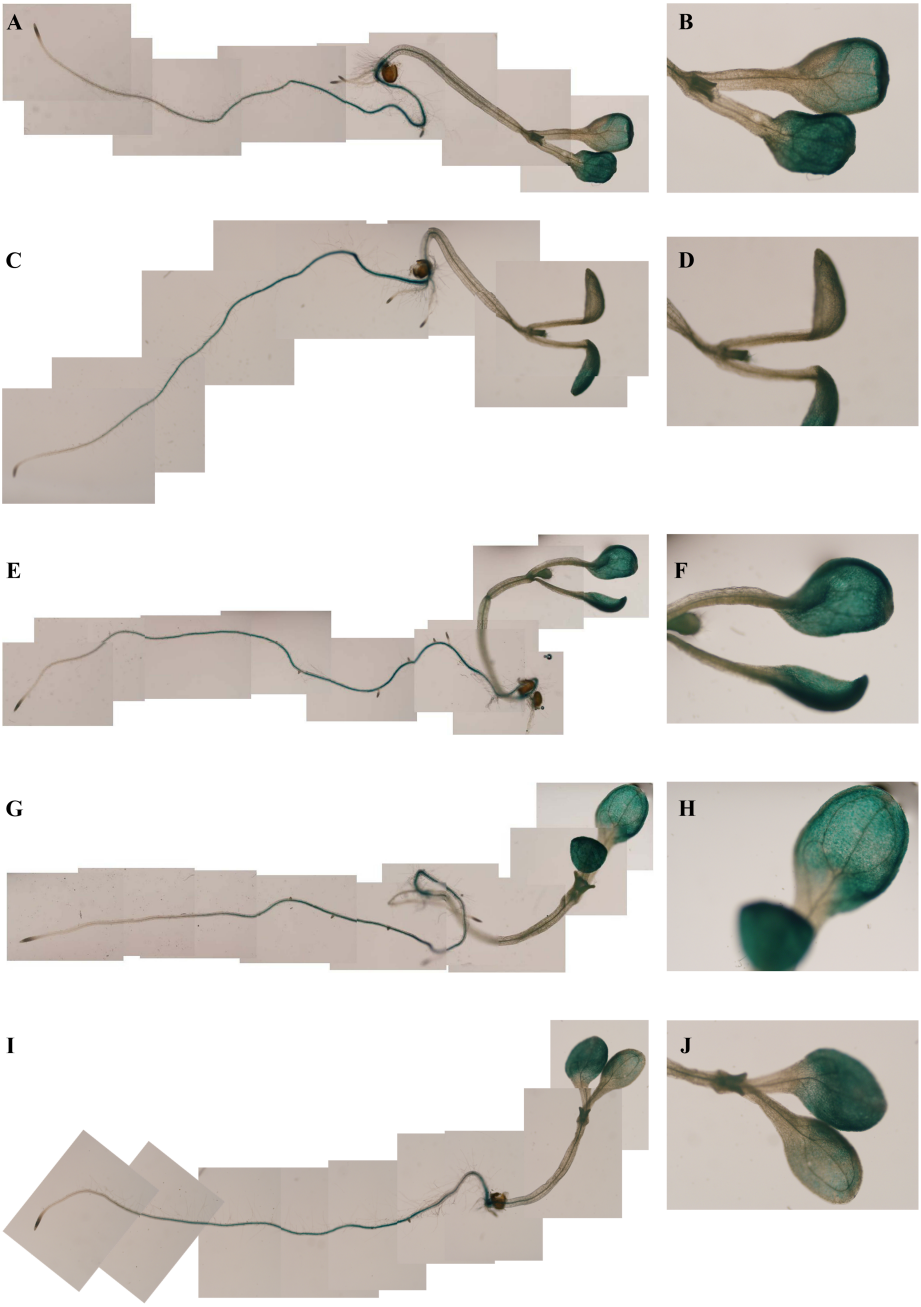
GUS histochemical staining in transgenic *Arabidopsis* under various stress treatments. GUS histochemical staining for each treatment was carried out in 25-day-old seedlings from each of three independent T3 transgenic *Arabidopsis* plants with the *PsDREB2* promoter. Control (A: seedling; B: leaves) seedlings were treated with distilled water. Transgenic seedlings were treated with 100 µmol ⋅ L^−1^ ABA (C: seedling; D: leaves), 300 mmol ⋅ L^−1^ NaCl (E: seedling; F: leaves), 10% PEG6000 (G: seedling; H: leaves), and 4 °C (I: seedling; J: leaves) for 2 h. The left line represents the total *Arabidopsis* plants, and the right line represents the leaves for each stress treatment.

**Figure 5:**
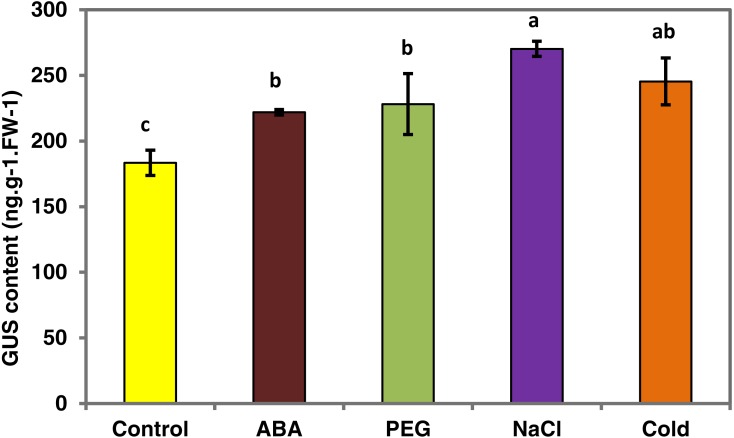
GUS activity of transgenic *Arabidopsis* in response to various stress treatments. The GUS activity for each treatment was measured in 25-day-old seedlings from each of three independent T3 transgenic *Arabidopsis* plants harboring the *PsDREB2* promoter. Transgenic seedlings were treated with 100 µmol ⋅ L^−1^ ABA, 300 mmol ⋅ L^−1^ NaCl, 10% PEG6000, and 4 °C for 2 h. Control seedlings were treated with distilled water. Data are presented as the means of three replicates with ±SDs shown by vertical bars. Different letters of a, b and c differ significantly by one-sided paired *t* test at *P* < 0.05.

**Figure 6:**
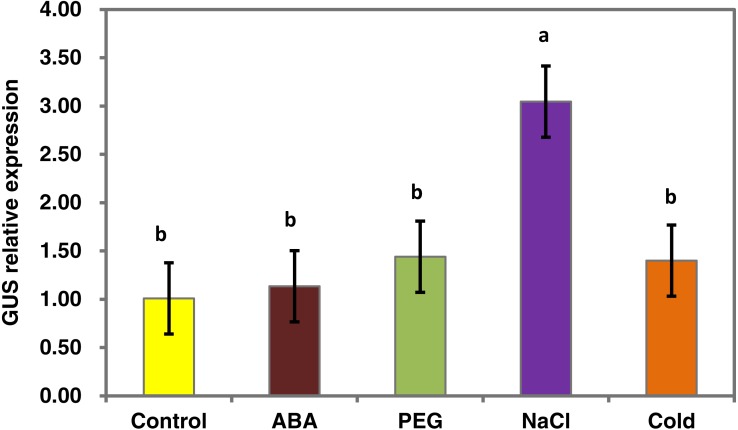
Real-time PCR analysis of the *GUS* gene in transgenic *Arabidopsis* in response to various stress treatments. The relative expression level of the *GUS* gene for each treatment was measured in 25-day-old seedlings from each of three independent T3 transgenic *Arabidopsis* plants with the *PsDREB2* promoter. Transgenic seedlings were treated with 100 µmol ⋅ L^−1^ ABA, 300 mmol ⋅ L^−1^ NaCl, 10% PEG6000, and 4 °C for 2 h. Control seedlings were treated with distilled water. Data are presented as the means of three replicates with ± SD shown by vertical bars. Different letters of a and b differ significantly by one-sided paired t test at *P* < 0.05.

To determine whether the *PsDREB2* promoter is environmentally regulated, 25-day-old T3 transgenic *Arabidopsis* plants were exposed to 100 µmol ⋅ L^−1^ ABA, 300 mmol ⋅ L^−1^ NaCl, 10% polyethylene glycol 6000 (PEG6000), and low temperature (4 °C) for 2 h. GUS activity was then tested. After exposure to different abiotic stresses, GUS activity was induced in all plants compared with the control. Figure 4 shows that after exposure to low temperature, ABA, PEG and NaCl, GUS staining had different degrees of deepening. GUS staining area in the root maturation zone increased, and the color deepened. However, in the root elongation zone and root tip, the staining did not deepen. All GUS staining in the leaves increased, especially after treatment with 10% PEG6000 and 300 mmol ⋅ L^−1^ NaCl. However, changes in GUS activity were difficult to observe by staining. Quantitative GUS activity and *GUS* gene expression levels were measured via ELISA and real-time PCR analysis, respectively. ELISA results revealed that GUS activity levels increased and were approximately 21.0%, 24.4%, 47.3%, and 33.8% higher than that in the control plants after exposure to 100 µmol ⋅ L^−1^ ABA, 300 mmol ⋅ L^−1^ NaCl, 10% PEG6000, and low temperature (4 °C), respectively (Fig. 5). Among these stresses, treatment with NaCl caused the most substantial changes in GUS activity, followed by cold and PEG treatment and then ABA treatment. There was no considerable difference in the GUS activity levels of plants exposed to ABA, PEG, and low temperature. Similar to the results of the GUS activity test, the expression level of the *GUS* gene was highest (3 times as much as the control) after salt treatment, followed by cold and PEG and then ABA (Fig. 6). There was no considerable difference among the three treatments. Salt stress enhanced the *GUS* expression level, whereas the other three stresses did not cause remarkable changes. This result was different from the GUS activity test results.

**Figure 7:**
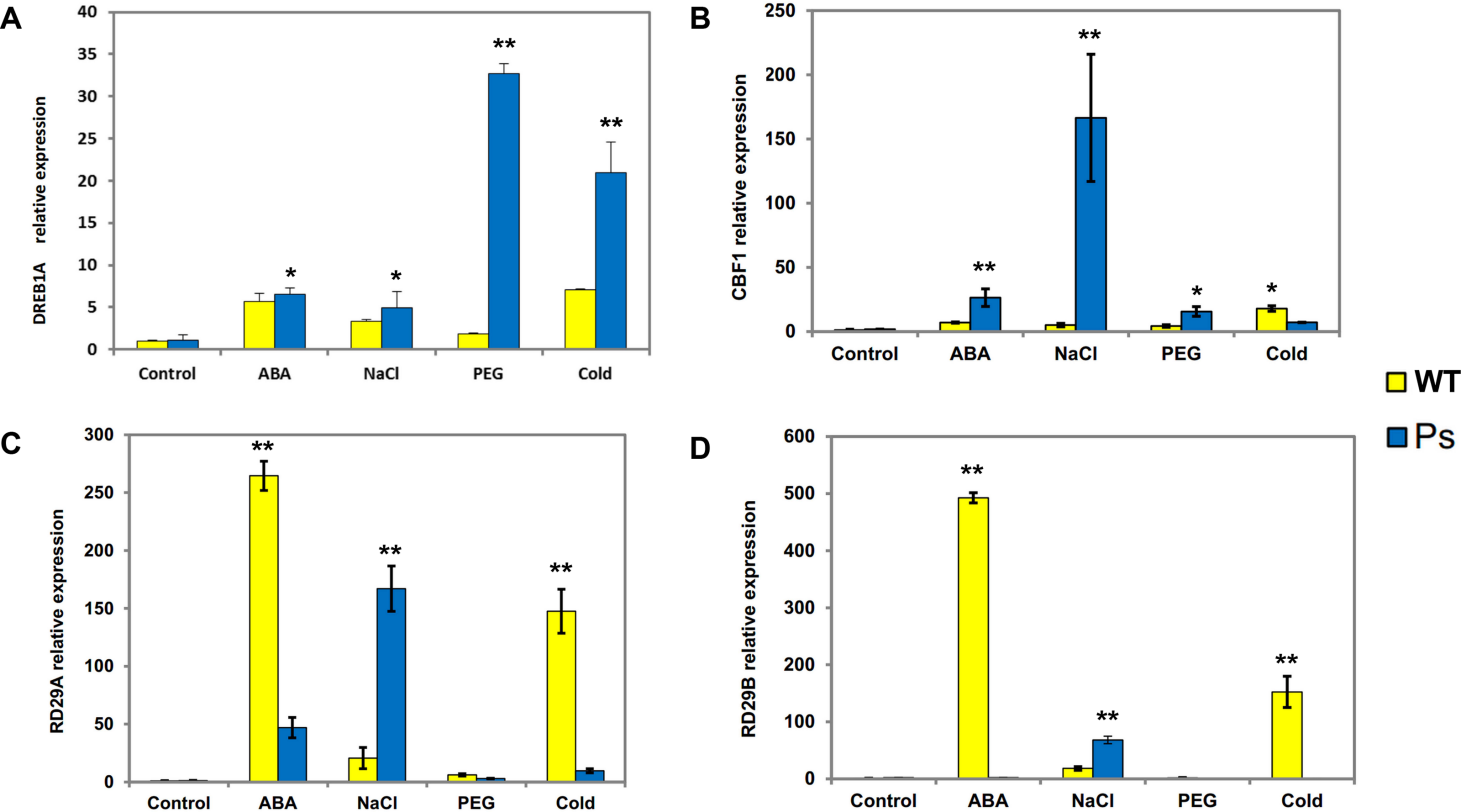
Expression patterns of *DREB1A*, *CBF1*, *RD29A*, *RD29B* in *Arabidopsis* plants with the *PsDREB2* promoter controlled by wild-type *Arabidopsis* plants under various stress treatments. WT, Wild-type *Arabidopsis* plants; Ps, Transgenic *Arabidopsis* plants with the *PsDREB2* promoter. The expression patterns of *DREB1A* (A), *CBF1* (B), *RD29A* (C) and *RD29B* (D) genes in wild-type and transgenic *Arabidopsis* plants were analyzed by qRT-PCR. Seedlings grown in 1/2 MS medium (25-day-old) were treated with 100 µmol ⋅ L^−1^ ABA, 300 mmol ⋅ L^−1^, 10% PEG6000, and 4 °C for 2 h. Control seedlings were treated with distilled water. Data are presented as the means of three replicates with ±SD shown by vertical bars. ^∗^*p* < 0.05; ^∗∗^*p* < 0.01.

### Expression patterns of stress-responsive genes in the *PsDREB2* promoter in transgenic *Arabidopsis* under abiotic stresses

*PsDREB2* transgenic tobacco plants have shown moderate tolerance to abiotic stresses, such as salt, ABA, drought, and low temperature (Liu et al., 2016). Hence, to determine whether the *PsDREB2* promoter influences the expression of stress-responsive genes in transgenic *Arabidopsis*, four related genes were analyzed via real-time PCR (qRT-PCR) (Fig. 7). The expression of the four stress-responsive genes (*DREB1A*, *CBF1*, *RD29A*, and *RD29B*) was investigated in both transgenic and WT *Arabidopsis* plants under ABA, salt, drought, and cold stresses. Different levels of expression of these four genes were detected in both transgenic and WT *Arabidopsis*. The expression levels of *DREB1A* in transgenic lines were higher than those in WT lines. The most substantial difference was observed under drought stress (10% PEG 6000 treatment). Except under cold stress, the transcripts of *CBF1* showed various degrees of upregulation in transgenic lines compared with WT plants, especially under salt stress (300 mmol ⋅ L^−1^ NaCl treatment). *RD29A* and *RD29B* exhibited similar expression patterns. Under salt stress, the expression levels of these two genes in transgenic *Arabidopsis* were higher than those in WT lines. Under ABA and cold stresses, the expression levels of *RD29A* and *RD29B* in transgenic lines were remarkably lower than those in WT plants. Under PEG stress, the expression levels of *RD29A* and *RD29B* were extremely low, in both transgenic and WT plants. Overall, all four genes showed extremely low expression levels, and there was no remarkable difference between the two types of *Arabidopsis* plants without any stress treatment.

## Discussion

*DREBs* are transcription factors identified in different plant species. These transcription factors induce the expression of the functional target genes involved in abiotic stresses. Until 2002, there were 14 *DREB* genes found in *Arabidopsis*, and these genes were categorized into two subclasses: *DREB1* and *DREB2* (Sakuma et al., 2006). Since then, other *DREB* genes, such as *DREB3*, *DREB4*, *DREB5* and *DREB6*, have also been found in *Arabidopsis*. *DREB1* genes typically regulate cold-responsive genes, whereas *DREB2* genes are responsible for responses to drought and salt stresses (Nakashima, Ito & Yamaguchi-Shinozaki, 2009). Some reports have shown that many of these gene functions overlap (Shinozaki & Yamaguchi-Shinozaki, 2000; Knight & Knight, 2001). The *PsDREB* gene of *P. suffruticosa* was cloned in our previous research. Through sequence alignment, we found that the *PsDREB* gene is highly homologous to other *DREB* genes (Liu et al., 2015). We also detected its response to drought, high-salt, ABA, and low-temperature stresses. The results revealed that PsDREB protein has strong binding and transcriptional activities. The overexpression of *PsDREB* enhanced the resistance of plants to abiotic stresses, especially under drought and high-salt treatments (Liu et al., 2016). The sensitivity of *PsDREB* in response to drought and high-salt stress indicated that the *PsDREB* transcription factor gene may belong to the *DREB2* subclass and play an important role in exposure to abiotic stresses. Combined with the results of the multiple sequence alignment, we identified the *PsDREB2* gene.

Promoter cis-element analysis is pivotal to studying the function of promoters. Currently, PLACE and PlantCARE databases are widely used in research on plant promoter components to analyze and predict the cis-elements that may exist in the promoter. A 2.2-kb promoter sequence of the *PsDREB2* transcription factor gene was cloned from the *P. suffruticosa* genome and analyzed via PLACE and PlantCARE. Bioinformatics analysis revealed that there were many TATA box and abundant CAAT box sequences in the *PsDREB2* gene promoter region. A typical promoter contains a TATA box and a CAAT box, whose function is related to transcription initiation. This information indicates that the *PsDREB2* promoter has typical promoter characteristics and functions. A typical promoter has some tissue-specific and stress-responsive *cis*-element sequences. In our study, only one tissue-specific *cis*-acting element required for meristem expression was present in the *PsDREB2* promoter. Furthermore, there were 22 putative *cis*-elements containing abiotic and biotic stress-responsive elements in the promoter region (Table 2) that were similar to the *DREB2* promoter in *A. thaliana*. These elements included two light-responsive elements (G Box and GT1 motif), four ABA-responsive sites (ABRE), a MeJA-responsive site (CGTCA motif), a GARE motif, an SA-responsive site (TCA motif), one ARE, a drought-responsive MYB-binding site (MBS) element, eight drought- and ABA-responsive MYB recognition sites, and a drought-, ABA- and cold-responsive MYC recognition site. We identified more than eight TATA boxes, five drought-responsive elements, one GARE and one ABRE in the *PsDREB2* promoter, although this species contained five fewer light-responsive elements and 1 less auxin-responsive element than the *AtDREB2* promoter. Combined with the results of our previous work (Liu et al., 2016), we concluded that the *PsDREB2* promoter is not only inducible but also tissue-specific. Previous studies have shown that the accumulation of stress-related gene expression products driven by a constitutive promoter generally enhances the tolerance of plants (Ito et al., 2006; Kasuga et al., 1999; Hsieh et al., 2002). However, persistent stress tolerance sometimes inhibits plant development and growth (Sinha, Williams & Hake, 1993; Kurek et al., 2002; Kanneganti & Gupta, 2008). Therefore, screening and cloning stress-inducible promoters are paramount to drive inducible tolerance genes. The promoter obtained in this study made important contributions to this work. To further identify the minimum promoter lengths for high activity and verify the functions of the components, subsequent experiments were performed using promoter deletion and mutational analyses (Sun et al., 2010; Chen et al., 2012; Niu et al., 2018). In addition, yeast-specific impurity assays, gel block electrophoretic mobility shift assay (EMSA) and DNase I footprinting were used to determine the specific location of cis-elements and to identify their interacting proteins (Chen et al., 2015; Li et al., 2017; Zhou et al., 2019).

The pBI121 transgenic *Arabidopsis* were grown to various stages and then subjected to histochemical staining and fluorometric analysis for GUS activity to study the expression patterns of *PsDREB2* in detail. GUS staining revealed that, except in the seeds, the *PsDREB2* promoter was expressed in different tissues and organs of *A.thaliana*, such as roots, stems, leaves, flowers and the seed pod (Fig. 2A).The characteristic of no expressionin seeds is similar to the *TsVP1* promoter reported in *Thellungiella halophila* (Sun et al., 2010) and the *GmPRP2* promoter in soybean (Chen et al., 2014). This special trait would be useful in applications of the *PsDREB2* promoter in genetic engineering with little concern about food safety. For example, the *PsDREB2* promoter can be applied in transgenic oil peony projects to produce safe edible oils. Histochemical staining for GUS activity was detected in *Arabidopsis* at different developmental and growth stages. In 5–25-day-old seedlings, GUS staining first appeared in the roots, followed by the leaves and stems. In tissues and organs, including flowers, trichomes, and veins, GUS activity was detected (Figs. 2B–2D).The quantitative expression of GUS staining was determined via fluorometric assay to monitor the spatiotemporal expression pattern of the *PsDREB2* promoter. Consistent with the qualitative test results, the highest expression level was observed in the roots, followed by the leaves and stems (Fig. 3). The *PsDREB2* promoter showed a root-preferential expression pattern in the vegetative growth stage of *Arabidopsis* plants. This finding was different from previous reports on the function of the *DREB2* promoter; *Arabidopsis DREB2C* was preferentially expressed in vascular tissues (Chen et al., 2012).This difference was attributed to differences in genetic background or experimental conditions. However, the *PsDREB2* promoter was similar to the *GmPRP2* promoter, which has root-preferential expression in transgenic *Arabidopsis* (Winicov et al., 2004; Chen et al., 2014). The similarity was probably due to the similar *cis*-acting elements and their interwork in the promoter region.

Abiotic and biotic stresses influenced the expression of *DREBs*. Evidence indicates that a variety of stresses, such as drought, salt, cold, heat, mannitol, methyl viologen, ABA, and SA, up- or downregulate the expression of *DREBs*. For example, *GmDREB3* can be induced by low temperature and drought (Sun et al., 2008). Wheat *TaDREB6* expression levels increased when subjected to drought, low temperature, ABA, SA, and NaCl (Li et al., 2011). *AtDREB1A* is drought-inducible in transgenic *Salvia miltiorrhiza* (Wei et al., 2016). *DREB* gene expression is diverse and complicated under favorable and unfavorable conditions. The diversity of the promoter may regulate the expression of downstream genes through many biological channels. In this study, the activity of the *PsDREB2* promoter was upregulated under ABA, NaCl, PEG, and cold stresses (Figs. 5 and 6). GUS activity and *GUS* gene expression in transgenic *Arabidopsis* were profound under salt stress, which agreed with the results of our previous research on *PsDREB* gene expression (Liu et al., 2016). This similarity in the results could be related to the abundant *cis*-acting elements in the −400 bp sequence (−2,164 to −1,765) and the −250 bp sequence (−584 to 234) of the promoter. The former region contains five MYB recognition sites responsive to drought and ABA signals and one MYC recognition site responsive to drought, ABA and cold signals. The latter region contains two light-responsive elements (GT1-motif), one *cis*-element involved in gibberellin responsiveness, one *cis*-element (MBS) associated with drought induction, and one SA-responsive element (TCA).These two regions were probably sufficient for the salt stress response because of their ability to defend against and respond to stress (Sun et al., 2010). These regions may also contain a new element that is crucial for responding to salt stress. Hence, further research on these regions is necessary. Identification of the key elements and proteins that play important roles in the regulation of this promoter revealed a salt-resistance mechanism through promoter deletion assay. The upregulation of GUS activity under PEG stress could be due to the MBS element and MYB and MYC recognition sites involved in drought inducibility. Upregulation under ABA stress could be due to the ABRE elements and MYB and MYC recognition sites involved in ABA inducibility. Upregulation under cold stress could be due to MYC recognition sites at −2,034 bp upstream of the promoter sequence that responded to cold signals. *DREB* promoters reported in other species also had low-temperature, high-salt, and ABA stress inducibility. The rice *DREB1B* promoter showed a distinct stress-specific induction pattern in response to NaCl, PEG, cold, ABA, and other abiotic and biotic stresses. This promoter had similar stress-related *cis*-elements in the promoter region (Gutha & Reddy, 2008).

Constitutive expression of the *PsDREB2* promoter in transgenic *Arabidopsis* plants upregulated the expression of stress-responsive genes, such as *DREB1A*, *CBF1*, *RD29A*, and *RD29B*, when treated with abiotic stress. The expression of these four genes was extremely low in *Arabidopsis* under unstressed conditions. After exposure to ABA, NaCl, PEG, and low-temperature stresses, the expression level of these genes increased remarkably. These four genes in transgenic *Arabidopsis* plants with the *PsDREB2* promoter, compared with the *35S* promoter, were all strongly induced under salt stress. Based on these results, we speculated that the *PsDREB2* promoter retains its responsiveness to salt. The ideal regulatory effect of transgene expression levels is difficult to determine due to the lack of available promoters. This study provided important insights into the promoter regions that control salt-specific expression. This promoter can be widely used in the transgenic engineering of salt-resistant traits in *P. suffruticosa* and other plants, especially pioneer greening plants that need to be cultivated in saline soil.

## Conclusions

In this study, we isolated and analyzed the *PsDREB2* promoter from *P. suffruticosa*. The *PsDREB2* promoter is a tissue-specific promoter that has GUS activity in roots, stems, leaves, flowers, and silique pods but not in the seeds. Furthermore, the promoter is responsive to abiotic stresses, such as drought, low temperature, ABA, and especially high salt. Our research provided a useful tissue-specific and stress-responsive promoter that may be used in food-safe resistant transgenic engineering. To reveal the minimal key element in the promoter region required to induce tissue- and stress-specific expression, further research by loss of function analysis is needed.

##  Supplemental Information

10.7717/peerj.7052/supp-1 Supplemental Information 1 Raw data for Figs. 3, 5, 6 and 7 Quantitative GUS activity data in transgenic Arabidopsis plants obtained from GUS ELISA Kit Fig. 3 data: Given data show the concentration in root, stem and leaf of transgenic T3 Arabidopsis plants with the PsDREB2 promoter. Re-1, Re-2 and Re-3 represent the three replicates.
Fig. 5 data: Given data show the concentration in different stresses of transgenic T3 Arabidopsis plants with the PsDREB2 promoter. Arabidopsis seedlings were treated with different stresses for 2 h. Control: distilled water; ABA: 100 *μ* mol.L-1ABA; PEG: 10% PEG6000; NaCl: 300 mmol ⋅ L^−1^ NaCl; Cold: 4  °C. avg: average values of three replicates; std: standard deviation of three replicates. 5%: difference significance data by one-sided paired t test at *P* < 0.05, different letters of a, b, and c differ significantly.
Relative gene expression data in transgenic Arabidopsis plants under different stresses detected by real- time PCR.
Fig. 6 data: Given data show the GUS gene expression level of T3 transgenic Arabidopsis plants with the PsDREB2 promoter under various stress treatments. Arabidopsis seedlings were treated with different stresses for 2 h. Control: distilled water; ABA: 100 μ mol.L-1ABA; PEG: 10% PEG6000; NaCl: 300 mmol ⋅ L^−1^ NaCl; Cold: 4 °C. std: standard deviation of three replicates. 5%: difference significance data by one-sided paired t test at *P* < 0.05, different letters of a, b differ significantly.
Fig. 7AD˜ data: Given data show DREB1A (A), CBF1 (B), RD29A (C) and RD29B (D) gene expression levels in Arabidopsis plants with the PsDREB2 promoter controlled by wild-type Arabidopsis plants under various stress treatments. WT: Wild-type *Arabidopsis* plants. Ps: Transgenic *Arabidopsis* plants with the *PsDREB2* promoter. Arabidopsis seedlings were treated with different stresses for 2 h. Control: distilled water; ABA: 100 μmol.L-1ABA; PEG: 10% PEG6000; NaCl: 300 mmol ⋅ L^−1^ NaCl; Cold: 4 °C. std: standard deviation of three replicates.

## Additional Information and Declarations

### Data Availability

The following information was supplied regarding data availability: The raw data is available as a Supplemental File.
